# Early detection of risks in child development in Spanish-speaking countries: content validity

**DOI:** 10.3389/fped.2025.1444104

**Published:** 2025-03-05

**Authors:** Luis Felipe Llanos, María Martínez de Anguita

**Affiliations:** ^1^ Faculty of Economics and Business, Universidad Anánuac Mexico, Huixquilucan de Degollado, Mexico; ^2^ Office of Policy and Modernization, Instituto de Políticas Públicas del Estado de México y sus Municipios, Toluca, Mexico; ^3^ High Performance Research Group FERSE, Universidad Francisco de Vitoria, Pozuelo de Alarcón, Madrid, Spain

**Keywords:** early detection, attention deficit, hyperactivity, autism, learning disorders

## Abstract

**Introduction:**

Early detection of developmental disorders like ADHD, ASD, and LD is critical for improving educational outcomes and enabling timely interventions. This study aimed to develop a reliable, practical screening scale for Spanish-speaking children entering primary education, addressing gaps in early identification within the region.

**Methods:**

In total, 151 items were identified. With a qualitative methodology and with the help of 18 specialists in child therapy from various Spanish-speaking countries, stabilized content validity. The analysis was articulated in its aspects of relevance.

**Results:**

The validation process identified 18 items with strong psychometric properties. These items demonstrated high levels of agreement among experts and strong content validity, forming the foundation for a culturally relevant screening tool. The scale is designed to identify developmental risks early and support timely interventions in educational and clinical settings.

**Discussion:**

The study underscores the importance of efficient screening tools for primary education, especially in regions with limited access to early childhood education. Future research will validate the scale in larger, diverse samples to ensure its reliability, establish cutoff points, and confirm its generalizability across Spanish-speaking contexts.

## 1 Introduction

This study aimed to identify commonly used early detection questionnaires for ADHD, ASD, and LD that primary school teachers can administer without extensive training. We compiled a list of relevant scales for each disorder, which were then evaluated by a committee of experts. To ensure content validity, we selected scales carefully to avoid omitting essential dimensions or excessively including instruments.

Early detection of developmental risks is crucial for identifying biological, psychological, and social factors, as well as for recognizing developmental and learning disorders at an early stage (1–4). Such detection supports the implementation of targeted intervention programs, and the development of neuropsychological guidelines tailored to children's needs (5). In Spain, the National Institute of Statistics (6) reported that 16% of cases involving neuronal disorders do not receive early specialist care. Teachers play a key role in the early identification and referral of such cases, as noted by Mateu and Sanahuja (7).

Programs aimed at families and teachers to foster children's socialization and prosocial behavior have shown benefits, including improved self-esteem and reduced disruptive behaviors, emotional issues, hyperactivity, and aggression (8, 9). This highlights the importance of understanding how parents and teachers can contribute and what actions they should take. Engaging teachers in early detection has demonstrated significant improvements in subsequent treatments (10). Thus, equipping educators to address the needs of students with behavioral disorders is critical (11).

Neuropsychological evaluation plays a vital role in identifying neurodevelopmental disorders (NDD), employing specialized tools tailored to the child while considering their family and school environments (12). According to the DSM-V (2013), NDDs are categorized as (1) genetic disorders, such as Down syndrome or Rett syndrome; (2) environmental factors, like fetal alcohol syndrome; and (3) multifactorial conditions, including ADHD, autism spectrum disorders (ASD), and learning disorders (LD), which encompass intellectual disabilities, communication disorders, motor disorders, and tic disorders (13, 14).

NDDs affect approximately 20% of children and adolescents, with 4%–5% experiencing severe conditions (15). These children often encounter challenges such as fatigue, boredom, low self-esteem, motor control difficulties, and difficulties understanding instructions, all of which contribute to stress (12). Furthermore, DSM-classified mental disorders affect an estimated 12% of the general population, though many mental health issues in individuals under 18 remain underdiagnosed (2, 15).

Among NDDs, learning disorders (LD) are the most prevalent, affecting approximately 10% of school-aged children (16). ADHD is the second most common, impacting 3%–6% of this population (15, 17–19). Comorbidities are frequent, with individuals often exhibiting symptoms of multiple disorders (20). For instance, children with ASD show a significantly higher prevalence of comorbid disorders compared to their non-ASD siblings (21).

### 1.1 ADHD and the school

ADHD is a polygenetic disorder with a neurological basis that chronically affects behavior, academic performance, and social relationships. According to the DSM-V criteria by the American Psychological Association (22), it primarily impacts three areas: attention, hyperactivity, and impulsivity. ADHD often co-occurs with other conditions, including oppositional defiant disorder, conduct disorder, depression, anxiety, and substance abuse. Due to its complexity, early diagnosis by medical, psychological, and educational professionals is essential (17).

Detection of ADHD typically involves neuropsychological evaluation and behavioral observation. Initial assessments often include psychometric tests using pencil-and-paper methods to evaluate attention and impulse control, establishing a baseline for subsequent interventions (23). ADHD symptoms generally emerge during preschool years and can significantly affect later academic performance. In Spain, there is a shortage of standardized scales adapted to the local language and culture for detecting ADHD in preschoolers, underscoring the importance of early detection for effective clinical and educational interventions (24).

Given its complexity, diagnosing ADHD requires a multidisciplinary approach. The process often begins with school-based detection through behavioral questionnaires and scales completed by parents and teachers. A diagnosis is confirmed when assessments converge, though discrepancies between evaluations are common and can complicate the process (25). Commonly used tools are behavior scales and questionnaires based on DSM-IV and DSM-V criteria, with training provided to parents and teachers to improve accuracy (26, 27).

### 1.2 ASD and the school

The diagnosis of autism spectrum disorder (ASD) is primarily clinical (28). The American Academy of Pediatrics recommends screening all young children at 9, 18, and 24–30 months to detect developmental delays (29). However, the complexity of ASD presents challenges for traditional psychometric instruments in accurately tracking developmental trajectories (12).

ASD assessments are generally recommended at 12 months, at 2 years, and again between 4 and 5 years. However, certain forms of ASD, such as Asperger syndrome, may not become evident until the child faces greater social demands, typically during the early years of primary school (30).

### 1.3 LD and the school

Learning disorders (LD) are neurological conditions that hinder children with normal intelligence from achieving academic success due to insufficient learning resources. These disorders affect skills such as reading, writing, arithmetic, and attention (31). LDs persist throughout life, with dyslexia and dyscalculia being the most common forms. Dyslexia, characterized by reading and writing difficulties, affects between 5% and 17% of the population (16).

Early detection and diagnosis facilitate the implementation of methodological intervention programs rather than content-based strategies (32). Proper guidance for families is essential, as addressing LD requires a multidisciplinary approach and collaboration among educational agents (16). This study focuses on students from various Spanish-speaking countries, with future research potentially extending findings to different cultural and linguistic contexts. However, as Griel and Elatia (33) suggest, changing the language of educational and psychological tests may interfere with results.

Dyscalculia, involving difficulties in mathematics, affects approximately 5% of the population. Notably, two-thirds of children with dyscalculia also present with another developmental disorder (16).

### 1.4 Debate between professional tests and early detection tools

There is considerable debate regarding the relevance of questionnaires and behavioral observation scales for detecting ADHD, given the extensive list of symptoms outlined in the DSM-V (26) and inconsistencies in results. School-administered questionnaires often reveal discrepancies between reports from teachers and family members (18). For example, in an ADHD screening test, interobserver agreement between parents and teachers was found to be low (Kappa = 0.28) (34). Similarly, neuropsychological tests conducted by professional examiners often fail to correlate with questionnaires completed by parents (35). Furthermore, neuropsychological tests alone may be insufficient to differentiate ADHD from other psychiatric disorders (36).

### 1.5 Early detection tools

Early Intervention (EI) aims to support children with developmental disorders or those at risk. According to the State Federation of Early Intervention Professional Associations (37), early detection is critical for maximizing these children's potential. EI is informed by theoretical models such as Bronfenbrenner's (38) general systems theory, Sameroff and Chandler's (39) transactional development approach, Guralnick's (40) evolutionary systems model, and King et al.'s (4) transdisciplinary theory. These models offer strategies, resources, and guidance for parents and environments to improve the functioning of both children and their families (41).

There is broad professional consensus that early detection and intervention significantly enhance overall prognosis, particularly in cases with a high risk of severe developmental outcomes (42).

### 1.6 Debate on the use of scales for teachers vs. parents

The natural diversity among individuals (43) presents challenges for teachers in accurately identifying students' needs. Teachers often easily recognize students with high cognitive abilities, focusing primarily on memorization and reproduction, but may undervalue students who exhibit creativity or defiance toward authority (43). Despite these difficulties, 15% of public-school students in the United States received special education services under the Individuals with Disabilities Education Act (IDEA) in the 2022–23 school year.

In Chile, attention and behavior questionnaires are more commonly used than neuropsychological tests, making them a prominent tool for identifying ADHD (44). Early detection questionnaires offer significant advantages in school settings: they are cost-effective and can be quickly administered by teachers (18). Research shows that teachers improve the accuracy and validity of their assessments with appropriate training (45, 46).

Educational institutions are increasingly providing support services during the early detection stages of learning disorders (47). The primary beneficiaries of these efforts are children who require special attention (1). Although symptoms vary across individuals, early detection remains essential to enable timely and effective interventions (48).

## 2 Methodology

To uphold the principle of content validity, the selection process ensured that the scales were neither too narrow—excluding important dimensions—nor excessively broad, including unnecessary instruments (49).

### 2.1 Instrument selection for early detection of ADHD

The selection of instruments for ADHD assessment was based on the scales identified in a study conducted in Chile by Carreño and Gatica (44). The authors identified the five most widely used questionnaires for ADHD evaluation: (a) Conners' Test (50), (b) Scale for the Assessment of Attention Deficit Hyperactivity Disorder (EDAH) (51), (c) Vanderbilt Assessment Rating Scale (VARS) for Parents and Teachers (52), (e) Child and Adolescent Disruptive Behavior Inventory (CADBI) – Teacher Report Version (53, 54), and (f) Young's ADHD Questionnaire (YAQ-I), informant version (36).

#### 2.1.1 The selected ADHD tools

**Teacher rating scale by Conners (CTRS-15).** The scale is one of the most widely used tools for identifying childhood behavior problems, particularly ADHD. It has demonstrated high sensitivity and specificity, making it effective in distinguishing children with ADHD from those without it (55). The revised 15-item version (CTRS-15) by Purpura and Lonigan (56) selects five items from each subscale of the original CTRS-R. This streamlined version reduces the time required for teachers to complete it while retaining its ability to identify behavior problems. The 15 items are divided into three categories: 5 for inattention, 5 for hyperactivity/impulsivity, and 5 for oppositional behavior. Purpura and Lonigan (56) found the CTRS-15 to be psychometrically comparable to the original scale, while Gerhardstein et al. (57) confirmed its criterion validity, showing significant correlations with other ADHD measures.

**Scale for the assessment of attention deficit hyperactivity disorder (EDAH).** The scale is commonly used for evaluating ADHD in primary school students, with teachers completing it in 5–10 min. It includes five items each for hyperactivity and inattention, with the remaining items assessing behavioral problems. The EDAH enables structured teacher observations of a child's usual behavior, which are then analyzed to provide a global score and three standardized subscales (51).

**Vanderbilt ADHD rating scales for parents and teachers (VARS).** The Scale includes two versions: the Teacher (VADTRS) and the Parent (VADPRS) scales, designed for children and adolescents aged 6–12 years. These scales assess ADHD symptoms and other related behaviors. The VADTRS subscales cover inattention (items 1–9), hyperactivity (items 10–18), and additional areas such as disruptive behavior, anxiety-depression, academic performance, and school conduct. The factorial structure allows separate use of subscales for ADHD, conduct disorder (CD), and anxiety-depression disorder (58).

**Child and adolescent disruptive behavior inventory (CADBI).** The scale evaluates children and adolescents aged 3–18 years, with ratings provided by parents and teachers (53). Studies have validated its reliability and factorial structure across diverse samples from Brazil, Chile, Nepal, South Korea, Spain, Thailand, and the United States (59). CADBI comprises three subscales: behavior toward adults, behavior toward peers, and activity level at school. Each subscale includes 9 items related to ADHD. Its 8-point response scale facilitates ease of use, with scores ranging from 1 (“never in the last month”) to 8 (“10 or more times per day”).

**Young ADHD questionnaire-I (YAQ-I).** The scale has two versions: the self-reported YAQ-S and the informant-reported YAQ-I. Both versions include four subscales covering attention, hyperactivity, impulsivity, and emotional problems, with the YAQ-I adding 8 items specifically related to emotional issues (60, 61). The YAQ-I has demonstrated strong internal consistency across its subscales (36). Although most studies have focused on the YAQ-S version in adult students, results for this tool have been positive (62).

#### 2.1.2 Excluded ADHD scales

Several notable scales were excluded despite their recognized utility in school settings. For instance, the SNAP-IV scale by Swanson, Nolan, and Pelham (63) was excluded due to significant correlations with the previously selected tools. Two studies demonstrated this overlap: a Brazilian study comparing SNAP-IV with Conners' Scale (64) and a Taipei study comparing SNAP-IV with both the Conners' and Vanderbilt Scales (65).

The Barkley ADHD Rating Scale (66), another robust instrument for assessing ADHD symptoms, was excluded due to potential cultural discrepancies in certain items or concepts. While it has shown convergent results with Conners' Rating Scale, ongoing research aims to improve its cultural sensitivity (67).

Other commonly used tests, such as the WISC-IV Working Memory Test (for intelligence), Continuous Performance Test (CPT), Five Digits Test (FDT), Stroop Test, and the Revised Perception of Differences Test (CARAS-R), were also excluded. These tools are more oriented toward neuropathological evaluations rather than early detection purposes (44).

### 2.2 Instrument selection for early detection of ASD

The selection of scales for early ASD detection prioritized practicality and minimal time requirements for primary care professionals. Zúñiga et al. (29) recommended two specific questionnaires for early ASD screening due to their ease of use, quick administration, and suitability for primary school teachers: a) the ASQ-3 (Ages and Stages Questionnaire) (68) and b) the PEDS (Parents' Evaluation of Developmental Status) (69).

#### 2.2.1 The selected ASD scales

**Ages and stages questionnaire (ASQ-3).** The scale validated by Squires and Bricker (70) for Latin populations in the USA and Chile, is designed for children aged 8–30 months. This developmental screening tool tracks progress using a parent-focused approach, making it user-friendly and the most widely used developmental screener. The ASQ-3 consists of 30 items divided into five subscales, each with six items: (a) Communication, (b) Gross Motor, (c) Fine Motor, (d) Problem Solving, and (e) Personal-Social. If a child's total score falls within a specific range, the test recommends further evaluation by a professional (68).

**Parents' evaluation of developmental Status (PEDS).** Glascoe (69, 71) developed the Parents' Evaluation of Developmental Status (PEDS) based on extensive research into the predictive value of parental concerns for identifying behavioral and developmental problems in children. The PEDS is a 10-item scale, including two open-ended questions, for children aged 0–8 years. It incorporates a “PEDS Interpretation Form”, which provides an algorithm to guide professionals in responding to test results. Glascoe demonstrated that parental concerns are strong predictors of developmental and behavioral issues, making the instrument highly reliable and standardized.

#### 2.2.2 Excluded ASD scales

The M-CHAT, a widely used international standard for early autism detection in young children, was excluded from this study. Hardy et al. (72) and Beecham, as cited in Kong et al. (73), reported a high correlation between the M-CHAT and the ASQ-3. Additionally, Schonhaut et al. (74) highlighted that the M-CHAT lacks items addressing socio-emotional aspects.

ADOS-2, another commonly used tool for ASD diagnosis, was also excluded. While it is highly effective, its administration requires extensive training and specialized expertise in autism, making it less practical for general early screening purposes (75).

### 2.3 Instrument selection for early detection of LD

The selection of instruments for assessing learning disorders (LD) focused on widely recommended tools for detecting dyslexia and dyscalculia. These include (a) PRODISCAT, recommended by Bosch et al. (76), (b) PRODISLEX, for their detection in Spanish in the different educational cycles (77), and (c) Detection of Difficulties in Mathematics (DDAMat), specifically designed to identify early signs of dyscalculia (78).

#### 2.3.1 The selected LD tools

**PRODISLEX.** The scale protocol for detecting and intervening in dyslexia during early childhood education assesses two language-related dimensions: Oral Comprehension and Expression (6 items), and Reading/Writing (27 items). Additionally, it evaluates areas such as mathematics, understanding of time, cognitive aspects, health, personality, and psychomotor coordination. Responses are recorded in a binary format (yes/no) (79).

**PRODISCAT.** Developed by the College of Speech Therapists, PRODISCAT is designed for the educational field to assist teachers in early dyslexia detection. It includes a general dimension and a specific dimension, with the latter focusing on: Literacy (15 items), and Other areas such as mathematics and school performance. Like PRODISLEX, PRODISCAT uses binary responses (yes/no) (80).

**Test for the detection of difficulties in the field of mathematics (DDAMat).** The scale consists of 10 items in Spanish, each with five response options: Never, Almost Never, Sometimes, Frequently, and Always. Based on teacher observations, it identifies potential difficulties in mathematics (78).

#### 2.3.2 Excluded LD scales

Several well-known tools were excluded due to their limitations in early screening for primary education settings: BADyG (Battery of Differential and General Aptitudes): This tool is widely used to assess multiple cognitive abilities, including those linked to learning, and is particularly effective in the differential diagnosis of dyslexia and dyscalculia. However, it was excluded for the following reasons, it requires extensive training in educational psychology or neuropsychology for proper administration and interpretation, and its application time of 60–90 min is impractical for early screening purposes (81).

PROLEC-R battery (revised reading processes). This instrument is highly regarded for evaluating reading processes and related cognitive skills. However, it is primarily a diagnostic tool rather than a screening instrument. Its exclusion was based on the need for specialized training in neuropsychology and speech therapy for accurate administration and interpretation, and its requirement for individual administration for each student, as detailed in its application manual (82).

### 2.4 Other transversal, developmental, or regional scales considered and discarded

Several scales with multicultural or regional orientations were reviewed but ultimately excluded for specific reasons. Below are examples of the scales considered.

**Child behavior checklist (CBCL)**. This questionnaire covers a wide range of emotional and behavioral problems and is adaptable to various cultural contexts. It has been standardized in multiple languages and is widely used in research, making it a classic tool in child behavior assessment. However, the CBCL was excluded due to its length (140 items for children aged 6–18 years), which limits its practicality for large-scale screening in primary school settings (83).

**Bayley scales of infant and toddler development (BSID)**. The BSID is well-known for assessing general developmental progress, including identifying developmental delays. However, it was excluded because it focuses on children under 42 months, which falls outside the target population of this study (84).

**Evaluación neuropsicológica infantil (ENI)**. The ENI is commonly used in Mexico for assessing specific learning disorders but has limited application in other Spanish-speaking countries and is considered outdated (85). Additionally, it requires advanced expertise in child neuropsychology for proper administration and interpretation, making it impractical for early screening purposes (86).

By excluding these tools, this research focused on instruments that are practical, broadly applicable, and suitable for early detection in primary school settings.

### 2.5 Items for validation process

To create the Early Detection Index of Risks in Child Development for Spanish-speaking countries, designed to identify potential developmental disorders in children entering primary education, items were compiled from the selected scales for ADHD, ASD, and LD. Table 1 summarizes the number of items included from each scale.

**Table 1:** Items from the scales selected.

Scale	ADHD	ASD	LD	Total
CTRS-15	15			15
EDAH	10			10
VARS	18			18
CADBI	9			9
YAQ-I	8			8
ASQ-3		28		28
PEDS		8		8
Prodislex			30	30
Prodiscat			15	15
DDAMat			10	10
Total	60	36	55	151

### 2.6 Expert committee

The recommendations of Muñiz (87) and Hernández-Sampieri et al. (88) were followed to ensure content validity, defined as an instrument's ability to accurately measure the intended constructs. Additionally, McGartland et al.'s (89) guidelines were adopted, which suggest involving 2–20 experts, with a minimum of 5 and at least two specializing in measurement and evaluation (90). Based on these criteria, 20 experts were invited to participate in the validation process.

#### 2.6.1 Expert selection process

Experts were selected through convenience sampling, leveraging the researchers' personal and professional networks, as well as their affiliated universities. This approach facilitated access to individuals with expertise in psychology, education, and related fields. Initially, over 30 experts were approached and informed about the project. Some declined due to professional commitments, while others chose to self-exclude, citing either doubts about the project's objectives or concerns about their qualifications to contribute. Ultimately, 18 experts agreed to participate, fulfilling the study's criteria for academic and professional relevance.

#### 2.6.2 Expert profiles

The final group of experts included: 10 psychologists, 2 educational psychologists, 1 child clinical psychologist, 1 pedagogue, 3 graduates in education, and 1 graduate in physical and technical sciences.

These professionals represented both public and private institutions, with 10 holding specialized degrees. Their professional experience averaged 14.7 years, with a minimum of 6 years.

### 2.7 Validation process

Each expert received a randomized list of 151 items without being informed of the scale from which each item originated. The experts were sensitized to the objective of the exercise and asked to classify each item, based on their expertise, as representing a typical behavior associated with one of the three studied disorders: ADHD, ASD, or LD. Experts were permitted to abstain from classifying an item if unsure. Importantly, no interaction or consultation between experts was allowed during the process.

#### 2.7.1 Content validity assessment

To determine item eligibility for inclusion in the tool, content validity was assessed using the Chi-square goodness-of-fit method, which compared experts' classifications with the theoretical diagnosis (percentage of agreement). This approach followed the steps outlined by Vargas and Hernández (17).

For each item item *i* (1 ≤ *i* ≤ 151), agreement between theory and practice was calculated using a variable *O*_*ij*_ (Observed)

• *O*_*ij*_ = 1: If the disorder identified by expert *i* matches the theoretical diagnosis of the item's source.
• *O*_*ij*_ = 0: If there is no match.
• *e*_*ij*_ = 1: Constant, representing the expected value under perfect theoretical agreement.

The Chi-square goodness-of-fit statistic *X*^2^ for each item *i*, with degrees of freedom df = *n*−1, was calculated using Equation (1).

(1) $$X^{2}(i)=\;\sum_{\;j=1}^{n}\frac{{\left(O_{ij}-1\right)}^{2}}{1}\;$$

#### 2.7.2 Item selection criteria

Items were selected based on the calculated *X*^2^ statistic and associated *p*-values:

• Items with *p*-value <0.05 were considered valid, indicating no significant evidence to reject them.
• Items with *p*-value ≥0.05 were excluded.

Additionally, experts rated the relevance of each item for early detection on a 0–4 scale: 4 = Essential, 3 = Desirable, 2 = Neutral, 1 = Not desirable, 0 = Not recommended.

The average relevance score across experts was used to prioritize no more than 8 non-dismissed items for each disorder, ensuring a concise tool. The 8-item limit was based on evidence that shorter scales reduce participant fatigue and improve response rates (91, 92).

Items that met theoretical agreement but lacked satisfactory consensus on relevance were also excluded.

#### 2.7.3 Final discrepancies and consensus Index

After statistical reduction and item selection, a second review was conducted to confirm the robustness of the proposed test. This review employed the consensus index method described by Perales (93). An index of consensus ≥0.80 was considered indicative of high content validity. This step ensured that the final items met stringent criteria for both statistical significance and expert agreement.

## 3 Results

### 3.1 Items derived from ADHD scales

From the five ADHD scales selected, a total of 60 items were compiled. After consulting the experts about which disorder each item was related to, only 5 items (8.3%) showed 100% agreement between theory and practice among all experts who provided opinions. Additionally, 24 items (40%) demonstrated over 80% agreement between theoretical assignment and expert evaluation. For the remaining items, there was a notable theoretical-practical discrepancy among the experts.

The 29 items with agreement levels exceeding 80% had a Chi-square statistic *X^2^* <2, corresponding to a *p*-value of 0.00. Subsequently, within this group, 8 items were selected based on having the highest average score for the variable “Relevance” (ranging from 3.38 to 3.56 on a scale of 0 to 4). Table 2 presents the 8 items selected for early detection of ADHD, ranked by their relevance as rated by the experts.

**Table 2:** Suggested items identified from early detection tests in ADHD.

Item in English	Item in Spanish	Item source	Advising experts	Consensus among experts"	*X* ^2^	*P*-value	Average relevance
1. Constantly moves, is restless.	Se mueve constantemente, es intranquilo	EDAH	18	16	2	0.000	3.56
2. Easily distracted, shows limited attention span.	Se distrae fácilmente, muestra escasa atención	EDAH	18	16	2	0.000	3.56
3. Exhibits excessive motor activity.	Tiene excesiva inquietud motora	EDAH	18	17	1	0.000	3.50
4. Restless, always alert and in motion.	Es inquieto, siempre despierto y en movimiento	CTRS-15	18	17	1	0.000	3.50
5. Answers impulsively, even before hearing the complete question.	Responde precipitadamente, incluso antes de escuchar la pregunta completa	VARS	18	18	0	0.000	3.44
6. Cannot stay still.	No puedo quedarse quieto	CTRS-15	18	18	0	0.000	3.38
7. Stands up in the classroom when expected to remain seated.	Se pone de pie en el aula cuando debiera permanecer sentado	VARS	18	16	2	0.000	3.38
8. Acts as if “driven by a motor” or seems “on the go” during classroom activities	Actúa como si “impulsado por un motor” o pareciera “en marcha” durante las actividades del aula	CADBI	18	16	2	0.000	3.38

Items ordered by the Average Relevance.

Three of the selected items were derived from the EDAH scale, two from the CTRS-15, two from the VARS, and one from the CABDI. Notably, no items from the YAQ-I scale ranked among the top 8. The highest-ranked item from this scale was in position 15, with a *p*-value of 0.00 and an average relevance score of 3.19, falling below the threshold set by the top 8 selected items.

### 3.2 Items derived from ASD scales

From the two ASD scales selected, a total of 36 items were compiled. After consulting the experts about which disorder each item was related to, no items achieved 100% agreement between theory and practice among all experts. Only 2 items (5.60%) demonstrated an agreement of 83.3% between theoretical assignment and expert evaluation. For the remaining items, there was a notable theoretical-practical discrepancy among the experts.

The 2 items with agreement levels exceeding 80% had a Chi-square statistic *X^2^* = 3, corresponding to a *p*-value of 0.00. Within this subset, the relevance scores for these two items were 2.69 and 2.56 (on a scale of 0–4). Table 3 presents the 2 items selected for early detection of ASD, ranked by their average relevance scores as rated by the experts.

**Table 3:** Suggested items identified from early detection tests in ASD.

Item in English	Item in Spanish	Item source	Advising experts	Consensus among experts"	*X* ^2^	*P*-value	Average relevance
1. Makes unusual sounds or noises when speaking.	Dice o emite sonidos al hablar	PEDS	18	15	3	0.000	2.69
2. Cannot walk on tiptoes for 4.5 m.	No puede caminar de puntillas 4.5 m	ASQ-3	18	15	3	0.000	2.56

Items ordered by the Average Relevance.

One item originated from the PEDS scale and the other from the ASQ-3 scale. Notably, the average relevance scores assigned by the experts for items assessing early detection of ASD ranged between 2 (neutral) and 3 (desirable). This trend may be attributed to the fact that ASD diagnosis is primarily clinical in nature (28).

### 3.3 Items derived from LD scales

From the three LD scales selected, a total of 55 items were compiled. After consulting the experts about which disorder each item was related to, no items achieved 100% agreement between theory and practice among all experts. However, 18 items (32.7%) demonstrated over 80% agreement between theoretical assignment and expert evaluation. For the remaining items, there was a notable theoretical-practical discrepancy among the experts.

The 18 items with agreement levels exceeding 80% had a Chi-square statistic *X^2^* <3, corresponding to a *p*-value of 0.00. Subsequently, within this subset, 8 items were selected based on their highest average scores for the variable “Relevance” (ranging between 3.25 and 3.50 on a scale of 0–4). Table 4 presents the 8 items selected for early detection of LD, ranked by their relevance as rated by the experts.

**Table 4:** Suggested items identified from early detection tests in LD.

Item in English	Item in Spanish	Item source	Advising experts	Consensus among experts’	*X* ^2^	*P*-value	Average relevance
1. Has difficulty with mental calculations.	Tiene dificultad para el cálculo mental	DDAMat	17	15	2	0.000	3.50
2. Exhibits reading difficulties.	Presenta dificultades de lectura	Prodislex	18	15	3	0.000	3.50
3. Has trouble handling numbers and mathematical symbols.	Tiene dificultad para manejar números y símbolos matemáticos	DDAMat	18	17	1	0.000	3.44
4. Struggles to name mathematical quantities, numbers, symbols, and establish relationships.	Tiene dificultad para nombrar cantidades matemáticas, números, símbolos y establecer relaciones	DDAMat	18	15	3	0.000	3.38
5. Shows less reading fluency compared to the class group.	Tiene poca fluidez lectora en comparación con el grupo de clase	Prodiscat	18	15	3	0.000	3.38
6. Finds it difficult to interpret arithmetic operations.	Tiene dificultad para interpretar operaciones aritméticas	DDAMat	18	17	1	0.000	3.31
7. Struggles with solving problems that involve a certain degree of logical-mathematical reasoning.	Tiene dificultad en la resolución de problemas que impliquen cierto grado de razonamiento lógico-matemático	DDAMat	18	16	2	0.000	3.25
8. Omits or adds letters, syllables, or words (omissions and additions)	Omite o añade letras, sílabas o palabras (omisiones y adiciones)	Prodislex	18	15	3	0.000	3.25

Items ordered by the average relevance.

Five items were derived from the DDAMat scale, two from the Prodislex, and one from the Prodiscat. Notably, the experts prioritized items related to mathematical learning over reading, selecting 5 items for the former and 3 items for the latter. This highlights the emphasis placed on detecting mathematical learning difficulties in early education.

## 4 Discussion

To promote early detection, it is essential for students entering primary education to undergo evaluations by teachers and other educational figures. The tests presented in this study are specifically designed for students attending school. While primary education is mandatory in Spanish-speaking countries, pre-primary education is not universally compulsory. However, some nations have made notable progress in this area.

Through the expert validation process, we identified 18 items from the 10 most widely used, validated, and reliable psychometric scales. These items exhibited strong psychometric properties in terms of relevance and importance, providing a solid foundation for developing an early screening tool. This tool holds significant potential for use in schools to refer children who may develop developmental disorders affecting educational performance in Spanish-speaking regions to specialists at an early stage.

The 18 experts reached a consensus to integrate into a single construct for early detection of developmental risks in schoolchildren: 8 items from the ADHD scales, 8 from the LD scales, and only 2 items from the ASD scales. This differentiation in preferences arises because ASD scales are typically designed for diagnosing children younger than primary school age, which is the target population of this tool. However, certain forms of ASD, such as Asperger syndrome, often manifest during primary education when social demands increase. Therefore, we decided to retain these two ASD items for inclusion in the project.

Once reliability tests are conducted, this item base could become a highly effective tool for the early detection of developmental risks in schoolchildren. This scale holds promise for identifying students with potential neurodevelopmental challenges, enabling timely and appropriate medical and educational support.

### 4.1 Limitations

One significant limitation of this research was securing expert collaboration. The time commitment required and the international scope of the project led many individuals to decline participation during the initial stages. Participants were carefully selected to ensure diverse roles and professional backgrounds that could contribute meaningfully to the study. However, identifying suitable participants and maintaining their involvement proved challenging.

Another limitation was the use of convenience sampling for the expert committee, which may introduce biases. Despite this, the diversity of the experts' profiles—including years of experience, areas of specialization, and representation from both public and private institutions—strengthens confidence in the robustness of the validation process.

However, subjectivity in expert judgments, lack of weighting for expertise, limited applicability to constructs with certain boundaries, and challenges in generalizing results indicate that, while this procedure is somewhat useful, it should be supplemented with reliability tests. Validating the scale with larger, diverse samples is necessary to ensure its reliability, establish scoring cutoff points, refine item wording and phrasing, provide clear administration guidelines, and confirm its generalizability across Spanish-speaking contexts, thereby offering a more comprehensive assessment of content validity.

### 4.2 Future avenues of research

We plan to implement the developed scale across a large, representative sample in Spanish-speaking countries, focusing on:

(a) Evaluating Reliability and Structural Validity: Conducting reliability assessments, including tests for convergent and discriminant validity, to measure internal consistency. Additionally, performing exploratory factor analysis (EFA) to examine the scale's dimensionality, ensuring alignment with the intended constructs.
(b) Establishing Cutoff Points for Categorical Classification: Comparing outcomes between experimental and control groups to identify the minimum scores that warrant professional evaluation. This approach aims to transform the one dimensional scale into a practical categorical tool suitable for educational settings.

By leveraging a substantial and diverse sample from Spanish-speaking populations, these analyses aim to confirm the scale's robustness and generalizability, ensuring its applicability across various national contexts.

## Data Availability

The raw data supporting the conclusions of this article will be made available by the authors, without undue reservation.

## References

[1] Fuller EA Kaiser AP. The effects of early intervention on social communication outcomes for children with autism spectrum disorder: a metaanalysis. J Autism Dev Disord. (2020) 50:1683–700. 10.1007/s10803-019-03927-z 30805766 PMC7350882

[2] Costello EJ Egger H Angold A. 10-year research update review: the epidemiology of child and adolescent psychiatric disorders: I. Methods and public health burden. J Am Acad Child Adolesc Psychiatry. (2005) 44:972–86. 10.1097/01.chi.0000172552.41596.6f 16175102

[3] Giambona P Ding Y Cho S Zhang C Shen Y. Parent perceptions of the effects of early intensive behavioral interventions for children with autism. Behav Sci. (2023) 13:1–19. 10.3390/bs13010045 PMC9855042 36661617

[4] King G Strachan D Tucker M Duwyn B Desserud S Shillington M. The application of a transdisciplinary model for early intervention services. Infants Young Child. (2009) 22:211–23. 10.1097/IYC.0b013e3181abe1c3

[5] Martínez ÁC Matamoros AM. Neuropsicología infantil del desarrollo: detección e intervención de trastornos en la infancia. Rev Iberoam Psicol. (2010) 3:59–68.

[6] National Institute of Statistics [INE]. (2008). Encuesta de Discapacidad, Autonomía Personal y Situaciones de Dependencia 2008. Encuestas de discapacidades. Available online at: https://www.ine.es/dyngs/INEbase/es/operacion.htm?c=Estadistica_C&cid=1254736176782&menu=resultados&idp=1254735573175#_tabs-125473619471 (Accessed February 06, 2025).

[7] Mateu L Sanahuja A. Evaluación e intervención en TDAH y TND:: un caso abordado en el contexto escolar. Rev Psicol Clin Ninos Adolesc. (2020) 7:52–8. 10.21134/rpcna.2020.07.1.7

[8] Sánchez VC González BM. Comportamiento prosocial y agresivo en niños: tratamiento conductual dirigido a padres y profesores. Acta Investig Psicol. (2017) 7:2691–703. 10.1016/j.aipprr.2017.03.005

[9] Wright R John L Duku E Burgos G Krygsman A Esposto C. After-School programs as a prosocial setting for bonding between peers. Child Youth Serv. (2010) 31:74–91. 10.1080/0145935X.2009.524461

[10] Keane K Evans RR. The potential for teacher-student relationships and the whole school, whole community, whole child model to mitigate adverse childhood experiences. J Sch Health. (2022) 92:504–13. 10.1111/josh.13154 35191030

[11] Rabadán JA Giménez AM. Detección e intervención en el aula de los trastornos de conducta. Rev Facul Educ. (2012) 15:185–212. 10.5944/educxx1.15.2.132

[12] Pérez C Ruíz Y. Evaluación neuropsicológica en niños con trastornos del neurodesarrollo. Rev Méd Clín Las Condes. (2022) 33:502–11. 10.1016/j.rmclc.2022.07.007

[13] Artigas J Narbona J. Trastornos del Neurodesarrollo. Barcelona: Sociedad Española de Neurología Pediátrica (SENEP) (2011).

[14] Petersen S Posner M. The attention system of the human brain: 20 years after. Ann Rev Neurosci. (2012) 21:73–89. 10.1146/annurev-neuro-062111-150525 PMC3413263 22524787

[15] Caraballa M Gago A Ares J del Rio M García C Goicoechea A Prevalencia de trastornos del neurodesarrollo, comportamiento y aprendizaje en atención Primaria. Anal Pediatr. (2018) 89:153–61. 10.1016/j.anpedi.2017.10.007 29169978

[16] Sans A Boix C Colomé R López A Sanguinetti A. Trastornos del aprendizaje. Pediatr Integ. (2012) 16:691–9.

[17] Villar IO. Impacto y detección de niños con trastorno por déficit de atención con hiperactividad. Educ Fut. (2004) 10:11–20.

[18] Salas S Gonzalez M Araya A Valencia M Oyarce S. Uso del test de rendimiento continuo de conners para diferenciar niños normales y con TDAH en Chile. Ter Psicol. (2017) 35:283–91. 10.4067/S0718-48082017000300283

[19] Fernández A López S Albert J Fernández L Calleja B López S. Trastorno por déficit de atención/hiperactividad: perspectiva desde el neurodesarrollo. Rev Neurol. (2017) 64:101–4. 10.33588/rn.64S01.2017005 28256695

[20] López I Förster J. Trastornos del neurodesarrollo: dónde estamos hoy y hacia dónde nos dirigimos. Rev Méd Clín Las Condes. (2022) 33:367–78. 10.1016/j.rmclc.2022.06.004

[21] Khachadourian V Mahjani B Sandin S Kolevzon A Buxbaum JD Reichenberg A Comorbidities in autism spectrum disorder and their etiologies. Transl Psychiatry. (2023) 13:1–7. 10.1038/s41398-023-02374-w 36841830 PMC9958310

[22] American Psychological Association [APA]. Diagnostic and Statistical Manual of Mental Disorders. DSM-V-TR® ed. Washington, DC: American Psychiatric Association (2013). 10.1176/appi.books.9780890425596

[23] Abad-Mas L Caloca-Català O Mulas F Ruiz-Andrés R. Comparación entre el diagnóstico del trastorno por déficit de atención/hiperactividad con el DSM-V y la valoración neuropsicológica de las funciones ejecutivas. Rev Neurol. (2017) 64:95–100. 10.33588/rn.64S01.2017011 28256694

[24] Marín JJ Borra MC Álvarez MJ Esperón CS. Desarrollo psicomotor y dificultades del aprendizaje en preescolares con probable trastorno por déficit de atención e hiperactividad estudio epidemiológico en navarra y la rioja. Neurología. (2017) 32:489–93. 10.1016/j.nrl.2016.02.009 27091681

[25] Delgado PS Bodoque AR Melia JM. Patrones diferenciales entre padres y profesorado en la detección de TDAH. Bordón. (2015) 67:143–66. 10.13042/Bordon.2015.67308

[26] Barkley RA. Avances en el diagnóstico y la subclasificación del trastorno por déficit de atención/hiperactividad: qué puede pasar en el futuro respecto al DSM-V. Rev Neurol. (2009) 48:101–6. 10.33588/rn.48S02.2009003 19280563

[27] Becker SP. Topical review: sluggish cognitive tempo: research findings and relevance for pediatric psychology. J Pediatr Psychol. (2013) 38:1051–7. 10.1093/jpepsy/jst058 23933842

[28] Idiazabal M Palau M Fernandez E Fierro G. Estudios neurofisiológicos en los trastornos del neurodesarrollo: potenciales evocados cognitivos. Medicina (Buenos Aires). (2023) 83:12–6. 36820476

[29] Zúñiga AH Balmaña N Salgado M. Los trastornos del espectro autista (TEA). Pediatr Integral. (2017) 21:92–108.

[30] Yunta JAM Baduell MP Salvadó BS Santasusana AV. Autismo: identificación e intervención temprana. Acta Neurol Colomb. (2006) 22:97–105.

[31] Mateos R. Dificultades de aprendizaje. Educ Psychol (Lond). (2009) 15:13–9.

[32] Rodríguez G Díaz V. Aprendizaje automático para detección de problemas cognitivos: una revisión de la literatura. Cien Tecnol. (2020) 20:9–22. 10.18682/cyt.vi0.4306

[33] Gierl M ElAtia S. Book review: adapting educational and psychological tests for cross-cultural assessment. Appl Psychol Meas. (2007) 31:74–8. 10.1177/0146621606288556

[34] Cornejo W Sánchez Y Gómez MD Ossio ÓH. Desempeño diagnóstico del cuestionario lista de síntomas del DSM IV para el tamizaje del trastorno de hiperactividad con éficit de atención (TDAH) en niños y adolescentes escolares. Acta Neurol Colomb. (2010) 26:133–41.

[35] Spano P Katz N DeLuco T Martin CO Tam H Montalto D Parent perceptions of pediatric neuropsychological evaluations: a systematic review. Child Neuropsychol. (2021) 27:922–48. 10.1080/09297049.2021.1908980 33847535

[36] Young S Gudjonsson GH. Neuropsychological correlates of the YAQ-S and YAQ-I self-and informant-reported ADHD symptomatology, emotional and social problems and delinquent behaviour. Br J Clin Psychol. (2005) 44:47–57. 10.1348/014466504X197769 15826343

[37] Early Intervention Professional Associations. Libro Blanco de la Atención Temprana. Madrid: Real Patronato (2000).

[38] Bronfenbrenner U. The Ecology of Human Development. Cambridge: Harvard U. Press (1979).

[39] Sameroff A Chandler MJ. Reproductive risk and the continuum of caretaking causality. In: Horowitz FD Hetherington EM Scarr-Salapatek S Siegel G, editors. Review of Child Development Research. Chicago: U. Chicago (1975). p. 187–244.

[40] Guralnick MJ. Why early intervention works: a systems perspective. Infants Young Child. (2011) 24:6–28. 10.1097/IYC.0b013e3182002cfe 21532932 PMC3083071

[41] Dunst CJ Trivette CM. Capacity-building family-systems intervention practices. J Fam Soc Work. (2009) 12:119–43. 10.1080/10522150802713322

[42] Busquets L Mirabell J Muñoz P Muriel N Español N Viloca L Detección precoz del trastorno del espectro autista durante el primer año de vida en la consulta pediátrica. Pediatr Integral. (2018) 22(105):e1–105.e6.

[43] Balongo E Mérida R. El clima de aula en los proyectos de trabajo. Crear ambientes de aprendizaje para incluir la diversidad infantil. Perf Educ. (2016) 38:146–62. 10.22201/iisue.24486167e.2016.152.57602

[44] Carreño M Gatica S. Determinación de la correlación en el uso del cuestionario de vanderbilt y la aplicación de pruebas neuropsicológicas para el diagnóstico del trastorno por déficit atencional. Revi Salud Públ Nutr. (2019) 18:1–7. 10.29105/respyn18.2-1

[45] Gagné F. Are teachers really poor talent detectors? Comments on Pegnato and Birch’s (1959) study of the effectiveness and efficiency of various identification techniques. Gift Child Q. (1994) 38:124–6. 10.1177/001698629403800305

[46] Jiménez C. Diagnóstico y Educación de los más Capaces. Madrid: UNED (2010).

[47] Romero RR Morales V Hernández A. Desarrollo de una herramienta para la evaluación de la calidad percibida en los centros de atención infantil temprana. Anal Psicol. (2015) 31:127–36. 10.6018/analesps.31.1.158191

[48] Biederman J. Attention-deficit/hyperactivity disorder: a selective overview. Biol Psychiatry. (2005) 57:1215–20. 10.1016/j.biopsych.2004.10.020 15949990

[49] Pérez L Carrera J García AM. Eficacia como constructo multidimensional en la determinación de estrategias de informatización empresarial. Ing. Rev Chil Ing. (2018) 26:354–69. 10.4067/S0718-33052018000200354

[50] Sims D Purpura D Lonigan C. The relation between inattentive and hyperactive/impulsive behaviors and early mathematics skills. J Atten Disord. (2012) 20:704–14. 10.1177/1087054712464390 23204060

[51] Farré A Narbona J. EDAH. Evaluación del Trastorno por Déficit de Atención con Hiperactividad. Madrid: TEA (2003).

[52] Langberg JM Vaughn AJ Brinkman WB Froehlich T Epstein JN. Clinical utility of the vanderbilt ADHD rating scale for ruling out comorbid learning disorders. Pediatrics. (2010) 126:e1033–8. 10.1542/peds.2010-1267 20937653 PMC2970758

[53] Shipp F Burns GL Desmul C. Construct validity of ADHD-IN, ADHD-HI, ODD toward adults, academic and social competence dimensions with teacher ratings of Thai adolescents: additional validity for the child and adolescent disruptive. J Psychopathol Behav Assess. (2010) 32:557–64. 10.1007/s10862-010-9185-6

[54] Lee S Burns GL Snell J McBurnett K. Validity of the sluggish cognitive tempo symptom dimension in children: sluggish cognitive tempo and ADHD-inattention as distinct symptom dimensions. J Abnorm Child Psychol. (2013) 42:7–19. 10.1007/s10802-013-9714-3 23325455

[55] Conners KC. Conners’ Rating Scales, Revised: User’s Manual. Toronto, Canada: Multi-Health Systems (1997). 10.1037/t81067-000

[56] Purpura D Lonigan C. Conners’ teacher rating scale for preschool children: a revised, brief, age specific measure. J Clin Child Adolesc Psychol. (2009) 38:263–72. 10.1080/15374410802698446 19283604 PMC3279732

[57] Gerhardstein RR Lonigan CJ Cukrowicz KC McGuffey JA. Factor structure of the Conners’ teacher rating scale-short form in a lowincome preschool sample. J Psychoeduc Assess. (2003) 21:223–43. 10.1177/073428290302100301

[58] Wolraich ML Feurer ID Hannah JN Baumgaertel A Theodora Y. Obtaining systematic teacher reports of disruptive behavior disorders utilizing DSM-IV. J Abnorm Child Psychol. (1998) 26:141–52. 10.1023/A:1022673906401 9634136

[59] Burns GL Preszler J Becker SP. Psychometric and normative information on the child and adolescent behavior inventory in a nationally representative sample of United States children. J Clin Child Adolesc Psychol. (2022) 51:443–52. 10.1080/15374416.2020.1852943 33428463 PMC8272731

[60] Young S. The YAQ-S and YAQ-I: the development of self and informant questionnaires reporting on current adult ADHD symptomatology, comorbid and associated problems. Pers Individ Dif. (2004) 36:1211–23. 10.1016/S0191-8869(03)00212-5

[61] Gudjonsson GH Sigurdsson JF Smari J Young S. The relationship between satisfaction with life, ADHD symptoms, and associated problems among university students. J Atten Disord. (2009) 12:507–15. 10.1177/1087054708323018 18716292

[62] Kim EK Suh SY H K. The reliability and validity of the Korean adult young ADHD questionnaire-self-report (K-YAQ-S) and young ADHD questionnaire-informant-report (K-YAQ-I). J Korean Neuropsychiatr Assoc. (2006) wpr-111724:578–87.

[63] Zhao D Zhang J. The effects of working memory training on attention deficit, adaptive and non-adaptive cognitive emotion regulation of Chinese children with attention deficit/hyperactivity disorder (ADHD). BMC Psychol. (2024) 12:1–12. 10.1186/s40359-023-01507-6 38317179 PMC10845547

[64] Rodoy MF Yamaguchi M Sandrini S Chassot A da Silva MA. Application of the SNAP-IV’s and Conners’ scales for screening of attention deficit-hyperactivity disorder. J Human Soc Sci. (2021) 26:49–53.

[65] Lin IC Chang SC Huang YJ Kuo TB Chiu HW. Distinguishing different types of attention deficit hyperactivity disorder in children using artificial neural network with clinical intelligent test. Front Psychol. (2023) 13:1067771. 10.3389/fpsyg.2022.1067771 36710799 PMC9875079

[66] Barkley RA Murphy KR Fischer M. ADHD in Adults: The Complete Guide to Understanding ADHD in Adults and Children. Atlanta, GA: Guilford Press (2008).

[67] NeuroLaunch. (2024). The Barkley ADHD Rating Scale: A Comprehensive Guide for Understanding and Assessing ADHD. August 4. Available online at: https://neurolaunch.com/barkley-adhd-rating-scale/?utm_source=chatgpt.com (Accessed February 06, 2025).

[68] Squires J Bricker D. Ages & Stages Questionnnaires. A ParentCompleted Child Monitoring System. Baltimor: Paul Brookes (2009).

[69] Glascoe FP. Parents’ concerns about children’s development: prescreening technique or screening test? Pediatrics. (1997) 99:522–8. 10.1542/peds.99.4.522 9093291

[70] Vargas C Hernández LM. Validez y confiabilidad del cuestionario “prácticas de cuidado que realizan consigo mismas las mujeres en el posparto”. Av Enferm. (2010) 28:96–106.

[71] Glascoe FP. Parents’ evaluation of developmental status: how well do parents’ concerns identify children with behavioral and emotional problems? Clin Pediatr (Phila). (2003) 42:133–8. 10.1177/000992280304200206 12659386

[72] Hardy S Haisley L Manning C Fein D. Can screening with the ages and stages questionnaire detect autism? J Dev Behav Pediatr. (2015) 36:536–43. 10.1097/DBP.0000000000000201 26348972 PMC4563819

[73] Kong X Zhu J Tian R Liu S Sherman HT Zhang X Early screening and risk factors of autism spectrum disorder in a large cohort of Chinese patients with Prader-Willi syndrome. Front Psychiatry. (2020) 11:594934. 10.3389/fpsyt.2020.594934 33329146 PMC7735061

[74] Schonhaut L Buron V Aguilera R Vargas L. Detección temprana de trastorno del espectro autista: revisión de las herramientas de tamizaje validadas en Chile. Andes Pediatr. (2023) 94:425–35. 10.32641/andespediatr.v94i4.4901 39808552

[75] Kamp-Becker I Albertowski K Becker J Ghahreman M Langmann A Mingebach T Diagnostic accuracy of the ADOS and ADOS-2 in clinical practice. Eur Child Adolesc Psychiatry. (2018) 27:1193–207. 10.1007/s00787-018-1143-y 29560529

[76] Bosch R Pagerols M Rivas C Sixto L Bricollé L Español-Martín G Neurodevelopmental disorders among Spanish school-age children: prevalence and sociodemographic correlates. Psychol Med. (2022) 52(14):3062–72. 10.1017/S0033291720005115 33436129

[77] Gatell A. Trastorno específico del aprendizaje. Pediatría Integral. (2022) 21:21–34.

[78] Gómez M. Discalculia:¿ la dislexia de los números?. detección e intervención. [Segovia tesis]. Universidad de Valladolid (2022). Available online at: https://uvadoc.uva.es/bitstream/handle/10324/54057/TFG-B.%201809.pdf?sequence=4&isAllowed=y

[79] Disfam (2010). Protocolos de dislexia PRODISLEX. (2010). Available online at: https://www.disfam.org/prodislex/ (Accessed February 06, 2025).

[80] Departament d’Ensenyament de la Generalitat de Catalunya. Protocolo de Detección y Actuación en la Dislexia. Ámbito Educativo. Cataluña: CLC (2012). Available online at: https://www.clc.cat/pdf/publicacions/documents/es/PRODISCAT%203o%204o%20ES.pdf

[81] Cortés B Yuste C Yuste D. BADyG E1. Manual Técnico (Batería de Aptitudes Diferenciales y Generales). Madrid: Editorial Ciencia de la Educación Preescolar & Especial (2011).

[82] Cuentos F Rodríguez B Ruano E Arribas D. PROLEC-R. Batería de Evaluación de los Procesos Lectores—revisada (b). Madrid: Hogrefe TEA Ediciones (2024). Available online at: https://web.teaediciones.com/Ejemplos/PROLEC-R_Extracto.pdf

[83] Achenbach TM Edelbrock C. Child behavior checklist. Burlington. (1991) 7:371–92.

[84] Klein-Radukic S Zmyj N. The predictive value of the cognitive scale of the Bayley scales of infant and toddler development-III. Cogn Dev. (2023) 65:101291. 10.1016/j.cogdev.2022.101291

[85] Ardila A Rosselli M Matute E Guajardo S. The influence of the parents’ educational level on the development of executive functions. Dev Neuropsychol. (2005) 28(1):539–60. 10.1207/s15326942dn2801_5 15992255

[86] Matute E Rosselli M Ardila A Ostrosky F. Evaluación Neuropsicológica Infantil—eNI (Child Neuropsychological Assessment). Mexico DF, Mexico: Manual Moderno/Universidad de Guadalajara/UNAM (2007).

[87] Muñiz J. Teoría Clásica de los Tests. Madrid: Pirámide (2008).

[88] Hernández-Sampieri R Fernández C Baptista P. Metodología de la Investigación. México: Editorial Mc. Graw Hill Interamericana Editores (2006).

[89] McGartland D Berg M Tebb SS Lee ES Rauch S. Objectifying content validity: conducting a content validity study in social work research. Soc Work Res. (2003) 27:94–104. 10.1093/swr/27.2.94

[90] Escobar J Cuervo A. Validez de contenido y juicio de expertos: una aproximación a su utilización. Av Med. (2008) 6:27–36.

[91] DeVellis RF Thorpe CT. Scale Development: Theory and Applications. Thousand Oaks, CA: Sage Publications (2021).

[92] Smith GT McCarthy DM Anderson KG. On the sins of short-form development. Psychol Assess. (2000) 12:102. 10.1037/1040-3590.12.1.102 10752369

[93] Perales RG. Diseño y construcción de un instrumento de evaluación de la competencia matemática: aplicabilidad práctica de un juicio de expertos. Ensaio. (2018) 26:347–72. 10.1590/s0104-40362018002601263

